# A qualitative Design and optimization of CIGS-based Solar Cells with Sn_2_S_3_ Back Surface Field: A plan for achieving 21.83 % efficiency

**DOI:** 10.1016/j.heliyon.2023.e22866

**Published:** 2023-11-25

**Authors:** Md. Ferdous Rahman, Md. Kamrul Hasan, Mithun Chowdhury, Md. Rasidul Islam, Md. Hafijur Rahman, Md. Atikur Rahman, Sheikh Rashel Al Ahmed, Abu Bakar Md. Ismail, Mongi Amami, M. Khalid Hossain, Gamil A.A.M. Al-Hazmi

**Affiliations:** a Advanced Energy Materials and Solar Cell Research Laboratory, Department of Electrical and Electronic Engineering, Begum Rokeya University, Rangpur, 5400, Bangladesh; b Solar Energy Laboratory, Department of Electrical and Electronic Engineering, University of Rajshahi, Rajshahi, 6205, Bangladesh; c Department of Electrical and Electronic Engineering, Bangamata Sheikh Fojilatunnesa Mujib Science & Technology University, Jamalpur, 2012, Bangladesh; d Department of Physics, Pabna University of Science and Technology, Pabna, 6600, Bangladesh; e Department of Electrical, Electronic and Communication Engineering, Pabna University of Science and Technology, Pabna, 6600, Bangladesh; f Department of Chemistry, College of Sciences, King Khalid University, P.O. Box 9004, Abha, Saudi Arabia; g Institute of Electronics, Atomic Energy Research Establishment, Bangladesh Atomic Energy Commission, Dhaka, 1349, Bangladesh

**Keywords:** Back Surface Field (BSF), Tin sulfide (Sn_2_S_3_), CIGS-Based solar cell, Non-toxic buffer layer, SCAPS-1D, Thin-film solar cell

## Abstract

Conventional Copper Indium Gallium Di Selenide (CIGS)-based solar cells are more efficient than second-generation technology based on hydrogenated amorphous silicon (a-Si: H) or cadmium telluride (CdTe). So, herein the photovoltaic (PV) performance of CIGS-based solar cells has been investigated numerically using SCAPS-1D solar simulator with different buffer layer and less expensive tin sulfide (Sn_2_S_3_) back-surface field (BSF). At first, three buffer layer such as cadmium sulfide (CdS), zinc selenide (ZnSe) and indium-doped zinc sulfide ZnS:In have been simulated with CIGS absorber without BSF due to optimized and non-toxic buffer. Then the optimized structure of Al/FTO/ZnS:In/CIGS/Ni is modified to become Al/FTO/ZnS:In/CIGS/Sn_2_S_3_/Ni by adding a Sn_2_S_3_ BSF to enhanced efficiency. The detailed analysis have been investigated is the influence of physical properties of each absorber and buffer on photovoltaic parameters including layer thickness, carrier doping concentration, bulk defect density, interface defect density. This study emphasizes investigating the reasons for the actual devices' poor performance and illustrates how each device's might vary open-circuit voltage (V_OC_), short-circuit current density (J_SC_), fill factor (FF), power conversion efficiency (PCE), and quantum efficiency (QE). The optimized structure offers outstanding power conversion efficiency (PCE) of 21.83 % with only 0.80 μm thick CIGS absorber. The proposed CIGS-based solar cell performs better than the previously reported conventional designs while also reducing CIGS thickness and cost.

## List of abbreviations

CIGS: Copper indium gallium selenide
CZTS: Copper zinc tin sulfide
CdS: Cadmium sulfide
ZnSe: Zinc selenide
ZnS:In: Indium doped zinc sulfide
Sn_2_S_3_: Tin Sulfide
FTO: Fluorine-doped Tin Oxide
BSF: Back surface field
V_OC_: Open circuit voltage
J_SC_: Short circuit current
FF: Fill factor
PCE: Power conversion efficiency
SD: Single-Donor
SA: Single-Acceptor
CdTe: Cadmium telluride
DOS: Density of States
TFSCs: Thin film solar cell
SC: Solar cell
PDT: post-deposition treatment
Eg: Band gap
PV: Photovoltaic
HTL: Hole transport layer
ETL: Electron transport layer
QE: Quantum efficiency
C–V: Capacitance-Voltage
SRH: Shockley read hall
VB: Valence band
CB: Conduction band

## 1 Introduction

The solar cell is a compulsory requirement for obtaining efficient, affluent, highly proficient, and low-cost electrical energy converted from sunlight [[1], [2], [3]]. At present, Copper Indium Gallium di-Selenide (CIGS) based thin-film solar cell (TFSC) is demanding due to cost-effectiveness and high-power conversion efficiency in the world energy society. The outdoor performance of CIGS is superb and the conversion efficiency is sufficiently high [[4], [5], [6]] and in CIGS-based solar cells, this chalcogenide material is the ideal choice for the p-type absorber layer [7]. Although CIGS has a high manufacturing cost, it outperforms other than thin-film technologies like a-Si: H and CdTe solar cells in terms of performance [[8], [9], [10]]. When the rare and costly materials like Gallium (Ga) and Indium (In) are used, the price is high. Due to this reason the CIGS layer would be as thin as possible [11]. The four elements such as copper (Cu), indium (In), gallium (Ga), and selenium (Se) are formed CIGS. According to reports, CIGS has an absorption coefficient of 10^5^ cm^−1^ and a band gap (Eg) of 1.1 eV [12,13]. Adjusting the Ga/In ratio on the CIGS absorber the band gap can be simply couturier from 1.02 eV to 1.69 eV [6,7].

CdS is the most usually used buffer layer in CdTe and CIGS SC [3,5]. However, Cadmium (Cd) is toxic and thus not an eco-friendly material [8,9]. CdS have a band gap of 2.4 eV [5] and absorb photons in light wavelengths from 270 to 520 nm [14]. Hence, it is essential to find alternative non-toxic buffer layers of comparatively higher band gap materials which will help in transmitting more photons to the absorber layer. For this purpose, various non-toxic and low-cost materials such as indium sulfide (In_2_S_3_), indium (In)-doped zinc sulfide (ZnS: In), and zinc selenide (ZnSe) can be applied. ZnS: In is a promising buffer layer for TFSCs [15,16]. ZnS: In with a band gap of 3.1 eV [17] gives better performance than CdS with better lattice matching [8,9]. Furthermore, the ZnSe shows optimum performance with a band gap of 2.9 eV [18]. Imam et al. improve the efficiency 21.15 % of CIGS TFSC replaced by the conventional i-ZnO layer with ZnO_1-x_S_x_ [19,20] and using different types of HTL boosting the efficiency above 25 % [[21], [22], [23]]. Reported experimental efficiencies of CIGS TFSC have been found to be 19.2 % [24], 19.9 % [25]. Moreover, the PCE of 22.6 % has been achieved with the CdS buffer layer using post-deposition treatment (PDT) [26]. To date, M. A. Green et al. shows the highest PCE of 23.6 % has been confirmed for CIGS-based TFSC [27]. There is also an article reporting with a PCE of 23.85 % for Cd-free CIGS TFSC. Using numerical analysis, theoretical efficiencies have found above 20 % [12,28]. Many researchers have been used the range of 1–2.5 μm of the CIGS layer as an ideal value [28]. Moreover, The PCE was obtained 21.3 % using ultra-thin CIGS/Si structure, where Si used as a second absorber layer with thickness of 1 μm [12,29]. Furthermore, the efficiency of 24.45 % has been achieved by adding a SnS BSF layer of thickness 0.3 μm [30]. On the other hand Imam et al. develop the efficiency of 26.1 % [31], 30 % [32], 35.6 % [33], and 37.1 % [34] with using different types of CIGS based tandem cell.

Literature review indicates that 1–4 μm thick CIGS are usually used as absorber layers also this analysis of works states that the PCE of CIGS-based TFSC is less than 28 % and 24 % for theoretical and experimental cases, respectively [35]. It is evident that as the CIGS absorber layer's thickness increases, solar cells will cost more. So, herein we proposed tin sulfide (Sn_2_S_3_) as a novel BSF for CIGS-based solar cells which reducing the CIGS absorber layer thickness and cost. Sn_2_S_3_ is used in TFSCs applications because of its band gap of 1.09 eV–1.11 eV and good stability [36]. Sn_2_S_3_ includes Sn (Tin) and S (Sulfer) which are both abundant on earth, therefore adding a thick layer of Sn_2_S_3_ can significantly reducing the cost of industrial CIGS solar cells. Sn_2_S_3_ is regarded as the ideal absorber material for solar cells from experimental and theoretical perspectives [36]. Actually, we have developed a non-toxic, affordable, and environmentally friendly semiconductor heterostructure for a CIGS-based solar cell. Furthermore, revealing the double junction solar cell's capacity to absorb the majority of incident solar photons, specifically the capacity to absorb longer-wavelength photons via effective BSF layer-mediated sub-band gap trail-state absorption. On the other hand, the modifications made to the traditional cell structure are primarily intended to address the issue of the ultrathin CIGS layers' insufficient absorption of the incident light. The employment of new materials as a BSF layer has resulted in additional alterations to the fundamental structure. On the other hand, the modifications made to the traditional cell structure are primarily intended to address the issue of the ultrathin CIGS layers' insufficient absorption of the incident light. The employment of new materials as a BSF layer has resulted in additional alterations to the fundamental structure. Numerous scholarly publications have suggested methods for enhancing the performance of ultrathin CIGS cells by assuming that the sole cause of the decline in efficiency would be optical losses. Almost all, the creative approaches looked at to get over this problem resulted in changes to the typical cell structure and the use of new BSF [37]. This work demonstrates that, when compared to alternative BSF-aided structures, Sn_2_S_3_ is the best BSF layer for CIGS solar cells [36]. After first simulating process is done then, we replaced the CdS buffer to make it Cd-free by inserting layers of X = ZnS: In and ZnSe, giving alternative novel FTO/X/CIGS/Sn_2_S_3_ heterojunction solar cells and then optimized the thicknesses of those buffer layers. The CIGS layer has been optimized by varying the thickness, doping concentration, defect density and interface defect density with various buffer layers. The thickness and doping concentration of the Sn_2_S_3_ BSF layer has also been optimized. We have optimized the back and front electrodes amongst various metals and finally Nickel (Ni) and Aluminum (Al) have been used as back and front electrodes in our proposed cells. At last, we have studied our three proposed solar cells for optimized structure. Our proposed CIGS-based solar cells give PCEs of 21.83 %, 21.72 % and 20.77 % with ZnS: In, CdS and ZnSe buffers, respectively, proclaiming superior performances to the CIGS-based solar cells reported earlier [38].

## 2 Device and simulation of proposed solar cells

**Fig. 1:**
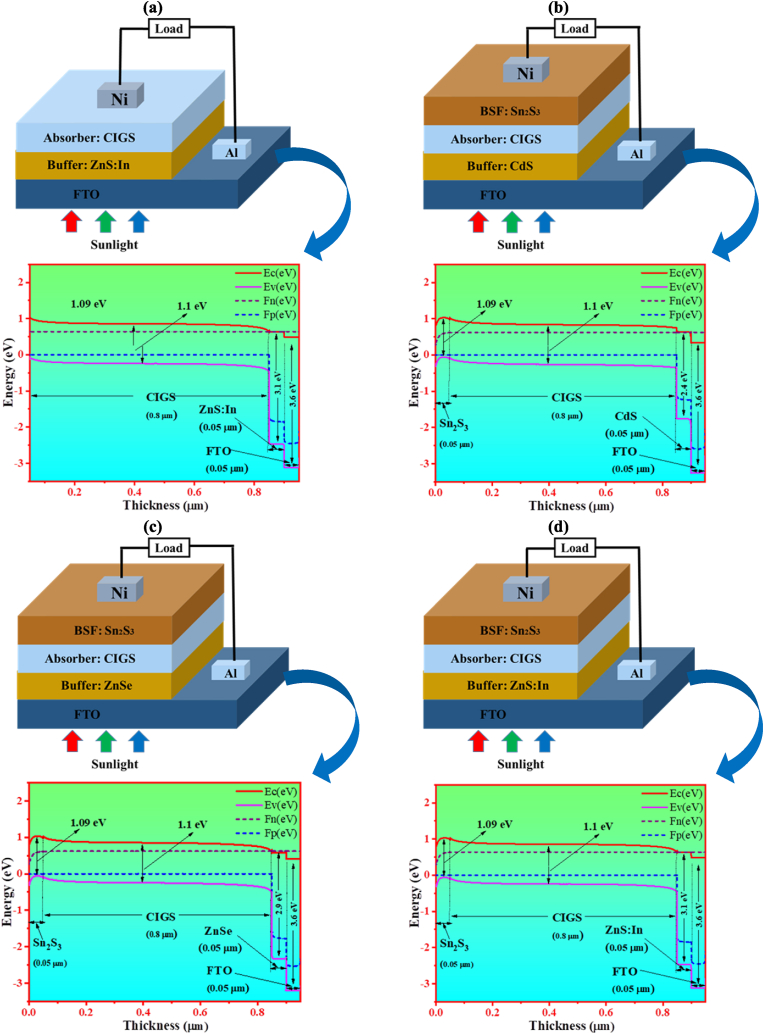
The proposed CIGS based structure and energy band diagram (a) without, and (b–d) with BSF.

Numerical designing approach plays an important task in thoughtful the device outputs to recognize the highly efficient PV cell. To scheme and evaluate polycrystalline TFSCs, the one-dimensional SCAPS-1 simulator has been taken as a promising tool in the scientific community of solar energy research [39]. The SCAPS-1D program can execute the simulation of a solar device including up to seven various semiconductor layers. This numerical method also quantitatively evaluates the heterojunction TFSCs by utilizing a group of parameters for numerous materials. In addition, it may effectively elucidate device characteristics assessed by other scientists with the entered likeness parameters of the employed films in the simulator and then the model achieves reliability. There are several research works have been reported in exploring the viewpoint of discovering heterojunction PV devices with the numerical simulations by the SCAPS-1D software [[40], [41], [42], [43], [44], [45]]. In their attempts, the PV performance parameters such as V_oc_, J_sc_, FF, efficiency, and spectral responses measured experimentally and theoretically are validated. In the earlier studies, it has been uncovered that the numerically obtained device outputs are exactly same to the experimental results, and thereby verifying the validity of the SCAPS-1D simulator [[43], [44], [45], [46], [47]]. Thus, the faithful SCAPS-1D tool can be efficiently expended to model and simulate heterojunction PV structures without any defeat of generality. In this analysis, the SCAPS-1D simulation software has been carried out to design and simulate the TFSC. The practically available material's parameters stated in the former investigations have been employed by considering all perception desirable to acquire best performance of the anticipated PV device structure. The optimized structure (Al/FTO/ZnS:In/CIGS/Ni) and energy band diagram is displayed in Fig. 1(a) without BSF. Furthermore three proposed solar cell structures (Al/FTO/X/CIGS/Ni) (X = CdS, ZnSe and ZnS:In) and energy band diagram are depicted in Fig. 1(b-d) with BSF. The p-type CIGS absorber has an Eg of 1.1 eV [12], the n-type CdS buffer layer has an Eg of 2.4 eV [48], and the Eg for the FTO window layer is 3.3 eV [48]. The back Nickel (Ni) contact layer is set with CIGS absorber layer (without BSF) and set with Sn_2_S_3_ BSF layer (with BSF). The traditional ZnO window layer has been altered by the FTO (SnO_2_: F) layer having *E*_*g*_ of 3.6 eV. There are three layers: a buffer layer made of n-type (*N*_*A*_ = 1 × 10^18^ cm^−3^) with thickness of 0.05 μm, an absorber layer made of p-type (*N*_*A*_ = 1 × 10^13^ cm^−3^) CIGS that is thickness of 0.8 μm, and Sn_2_S_3_ is used as a BSF of p^+^-type (*N*_*A*_ = 1 × 10^18^ cm^−3^) that is thickness of 0.05 μm in this proposed solar cell structure. As the front and back grid contact Aluminum (Al) and Nickel (Ni) were used with work function (WF) of 4.2 eV and 5.04 eV, respectively [8]. Due to the construction of solar cells, the work function is varied from 4 eV to 5 eV, and the results demonstrate that as the work function value developed, the efficiency also increased because a more significant work function value enhances band alignment of the rear surface. Due to a considerable energy-level disparity at the absorber metal contact, which can result in the formation of a Schottky junction and a decrease in device efficiency, the device performance can be negatively impacted below 4 eV. The greatest efficiency was attained because the work function was above 5 eV [49,50].

The absorber layer, composed of copper, indium, gallium, and selenium, absorbs photons to create electron-hole pairs. While the valence band is full with electrons, the conduction band is empty. The energy gap between them, or band gap, makes it possible to absorb photons with the required amount of energy. The gradient of the band gap and the fluctuations in the work functions cause the absorber layer to develop an intrinsic electric field. This field is used to separate and gather the electron-hole pairs that photons create. A p-n junction frequently forms in the absorber layer, which facilitates the separation and transportation of charge carriers. While the front contact serves as the hole collector, allowing for light transmission, the back contact, or a transparent conductive oxide layer, functions as an electron collector [51].

At first we proposed the cell structure FTO/CdS/CIGS/Sn_2_S_3_ which comprises toxic buffer layer of CdS. So, we have replaced the toxic CdS buffer of the baseline case with various non-toxic buffer layers such as ZnSe and ZnS: In. ZnSe. The proposed Cd-free CIGS structure contains with 0.8 μm thick absorber, 0.05 μm thick Sn_2_S_3_ BSF grown on a back Nickel (Ni) contact layer. Table 1 shows the input parameters used for simulation at 300 K. Entire simulation was carried out under global air mass AM 1.5G spectrum at a one sun illumination of 100 mW/cm^2^.

**Table 1:** Provides the simulation input values for the solar cell's layer's parameters.

Parameter (unit)	FTO [52]	CIGS [2,5,30,31]	Sn_2_S_3_ [36]	CdS [5,31,32]	ZnSe [8,14,17]	ZnS: In [9,33,34]
Thickness (μm)	0.05	0.8*	0.05	0.05	0.05	0.05
Band gap (eV)	3.6	1.1	1.09	2.4	2.9	3.1
Electron affinity (eV)	4	4.2	4.26	4.4	4.1	4.13
Dielectric permittivity	9	13.6	12.50	10	10	8.2
CB effective DOS (cm^−3^)	2.2 × 10^18^	2.2 × 10^18^	1.0 × 10^19^	2.2 × 10^18^	1.5 × 10^18^	6.35 × 10^18^
VB effective DOS (cm^−3^)	1.8 × 10^19^	1.8 × 10^19^	1.0 × 10^19^	1.8 × 10^19^	1.8 × 10^19^	6.03 × 10^19^
Electron thermal velocity (cm/s)	10^7^	10^7^	10^7^	10^7^	10^7^	10^7^
Hole thermal velocity (cm/s)	10^7^	10^7^	10^7^	10^7^	10^7^	10^7^
Electron mobility (cm^2^/V-s)	100	100	25	100	50	250
Hole mobility (cm^2^/V-s)	25	25	100	25	20	40
Donor density *N*_*D*_ (cm^−3^)	10^19^	0	0	10^18^	10^18^	10^18^
Acceptor density *N*_*A*_ (cm^−3^)	0	1 × 10^13^*	1 × 10^18^*	0	0	0
Defect type	SA	SD	SD	SA	SA	SA
Defect density (cm^−3^)	10^15^	10^14^*	10^15^	10^15^	10^15^	10^15^

Note: * is a variable field.

The band alignment reveals the amount of current flowing via the hetero-junction. The suggested cell's energy band diagram is also shown in Fig. 1 (a-d) utilizing simulation-derived data from the “SCAPS Energy Band Panel.” Each material's bandgap and layer thickness are obvious. This illustration illustrates how our suggested solar cell would be able to deliver the necessary results of an effective one.

## 3 Results and discussion

### 3.1 Different buffer layers study

**Fig. 2:**
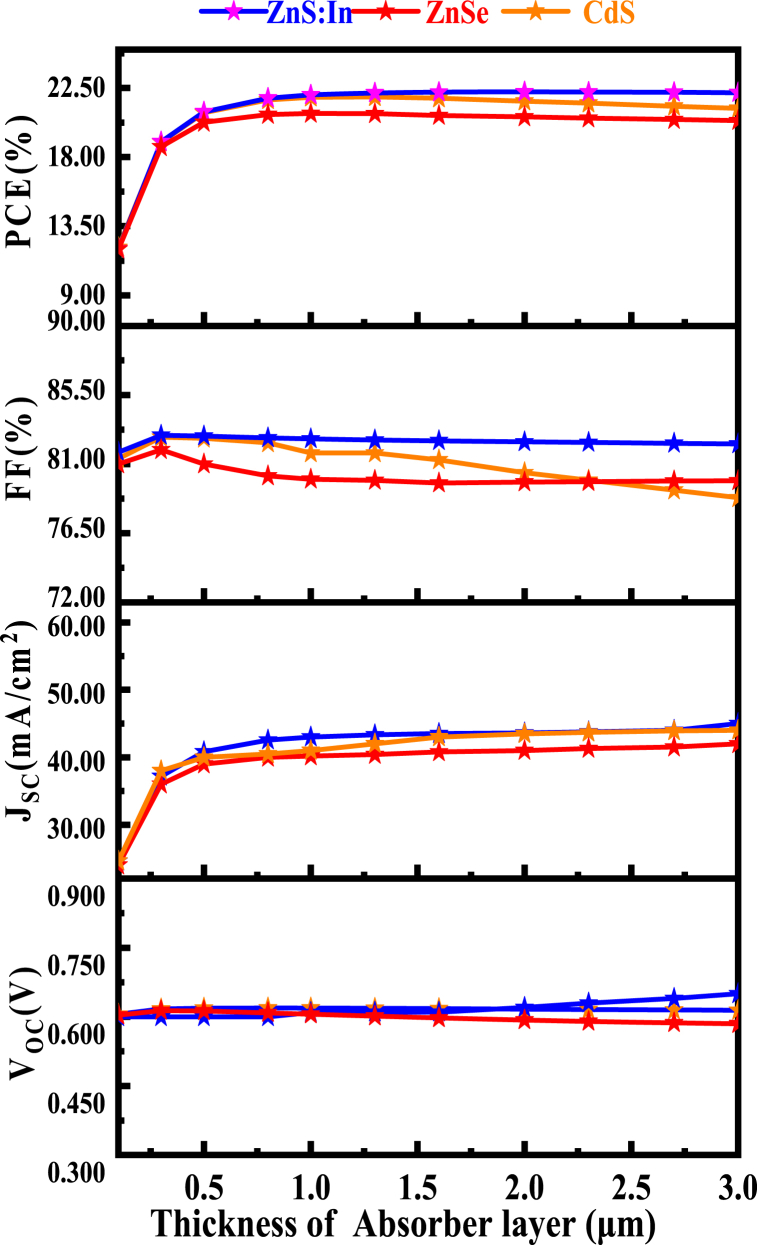
Comparison impact of three buffer layers with absorber thickness with BSF.

The comparison impact of three buffer layers with absorber thickness with BSF layer investigation of CIGS solar cell is shown in Fig. 2. At first we simulated proposed CIGS solar cell with toxic CdS buffer and we have replaced the toxic CdS buffer of the baseline case with various non-toxic buffer such as ZnSe and ZnS:In using the same thickness, doping, and defect densities. Furthermore it has been found that ZnSe and ZnS:In are the best alternative buffer layers for the CIGS solar cell. Table 2 summarizes the output parameters of the solar cell with different buffer layers. From Table 2, it is seen that all three-buffer layers give almost the same short-circuit current and open-circuit voltage.

**Table 2:** Photovoltaic parameters of the solar cell with different buffer layers.

S/N	Buffer layer	V_OC_ (V)	J_SC_ (mA/cm^2^)	FF (%)	η (%)
1	CdS	0.6194	42.56	82.38	**21.72**
2	ZnSe	0.6086	42.54	80.22	**20.77**
3	ZnS:In	0.6202	42.55	82.71	**21.83 (Optimized)**

But comparatively higher values of PCE and FF have been attained by ZnS:In buffer layers. The efficiency improvement may also be supported by determining the lattice mismatch which discuss in details in this manuscript in the section of 4.1.3 [53]. Hence, it can be concluded that ZnS:In is the appropriate alternative buffer layers for CIGS solar cell.

#### 3.1.1 Absorber layer (CIGS) thickness variation effect

Case-I Without BSF (Sn₂S₃)

**Fig. 3:**
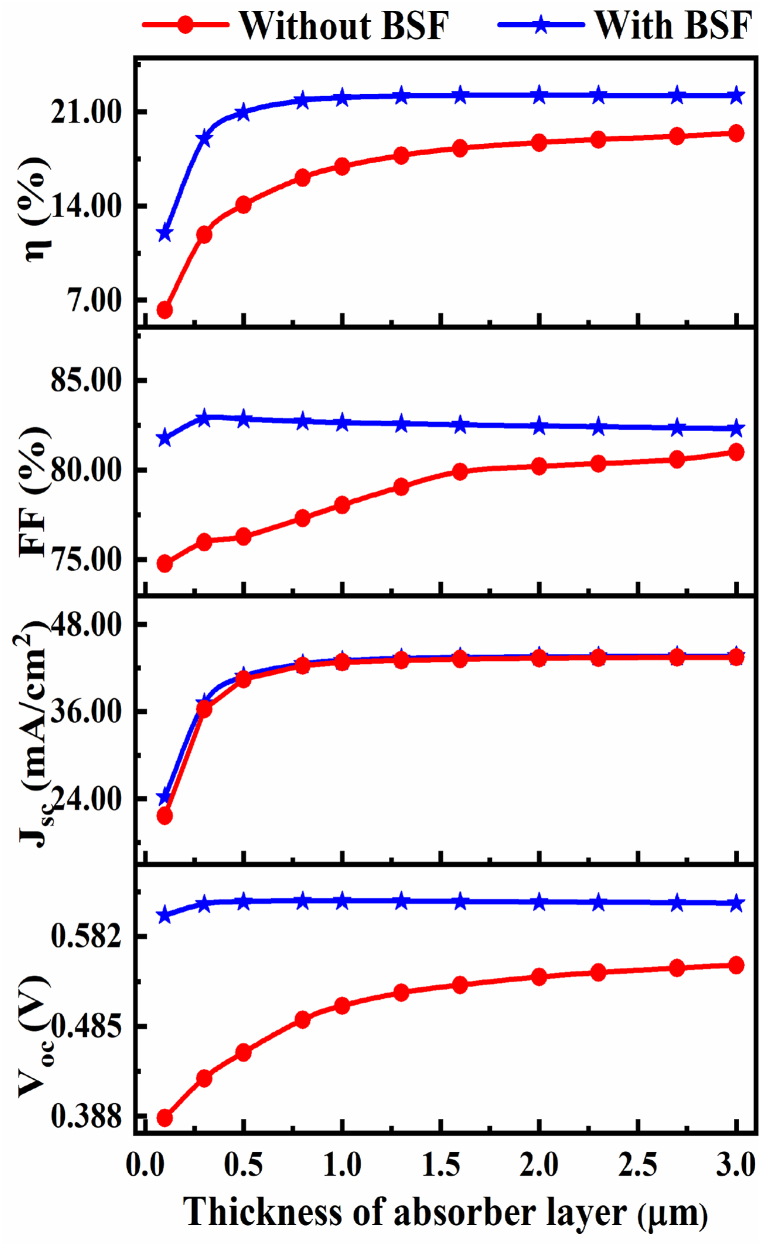
The impact of changing CIGS absorber thickness with and without BSF of optimized buffer ZnS:In.

The impact of the CIGS absorber layer thickness on the PV performance of the optimized cell is depicted in Fig. 3 with and without BSF. The CIGS absorber layer's thickness was changed from 0.1 μm to 3 μm for our optimized cell FTO/ZnS:In/CIGS and in this time, all other optimized value was constant in Table .1 with temperature 300 K. Furthermore single sunlight spectrum was chosen performed in this investigation.

When the thickness of the CIGS absorber layer growths the major portion of photons from the incident solar power are absorbed due to this circumstance directs rising efficiency (η) [54]. Efficiency ranges was varied from (6.25–19.40) % for absorber thickness (0.1–3.0) μm. The CIGS cell shows FF is 77.30 %, V_OC_ is 0.4919 V, J_SC_ is 42.33 mA/cm^2^ and η is 16.09 without BSF for optimized absorber thickness of 0.80 μm. Fig. 3 illustrates how efficiency rises with thickness and also a correlation is shown between the V_OC_ and J_SC_.

Case-II With BSF (Sn₂S₃)

The high doping impurity carrier concentration at the rear side of the structure drives minor charge carriers (electrons) away from the high recombination back metallic contact layer. The BSF layer is added with active absorber layer in the optimized structure that has a greater doping concentration. The BSF layer in the current study is positioned attached to the CIGS absorber layer and has a doping density of 1 × 10^18^ cm^−3^. The contact between the p^+^ type Sn₂S₃ and the p-type CIGS produce an electric field. These electric fields form a barrier that prevents the minority carriers from flowing to the rear surface. The Sn₂S₃ BSF layer will increase the short circuit current while simultaneously reducing the dark current by reflecting the minority carriers. The BSF layer minimizes surface recombination speed, improving the performance of solar cells [53].

At first we changed the thickness of the Sn₂S₃ BSF from 0.01 to 1 μm and all other optimized value is constant in Table .1. After a preliminary analysis, we discovered that cell efficiency stabilized in the region from 0.05 μm to 1 μm. As a result, Sn₂S₃ BSF layer was tuned to be 0.05 μm thin. However, the optimized CIGS solar cell provide an efficiency is 21.83 %, J_SC_ is 42.55 mA/cm^2^, V_OC_ is 0.62 V and FF is 82.71 at the same thickness of 0.8 μm. The efficiency is greatly improved while maintaining the same absorber layer thickness. The main contention is that both Sn₂S₃ and CIGS serve as absorbers for solar cells can be explored, the Sn₂S₃ layer assisting in increasing photon energy absorption within the cell. More electron-hole pairs can be produced as a result of the ability to absorb a large number of photons [55].

High-cost components used in CIGS-based solar cells include Indium and Gallium [9]. Therefore, it is not advisable to produce absorbers with significant CIGS thickness. Thus, we can reduce the use of Ga and In materials by reducing the thickness of the CIGS. In the end, it will result in decreased material manufacturing costs. The cost-effective new architecture solar cell has a higher efficiency than earlier models. Our research can help manufacturers of CIGS solar cells make decisions that are in their best interests. In this study, a slightly higher Jsc is observed for the CIGS solar cell with BSF layer than the cell without BSF layer at thin absorber layer in Fig. 3. The development of dark current due to insignificant built-in potential at the absorber/BSF junction may be the possible reason for the insufficient current density by adding BSF layer in the heterojunction CIGS solar cell.

However, the CIGS cell shows FF of 77.30 % with V_OC_ of 0.4919 V, J_SC_ of 42.33 mA/cm^2^, and η of 16.09 % without BSF for optimized absorber thickness of 0.80 μm. On the other hand, the optimized CIGS solar cell provides an efficiency of 21.83 % including J_SC_ of 42.55 mA/cm^2^, V_OC_ of 0.62 V, and FF of 82.71 % at the same thickness of 0.8 μm. It is revealed that the efficiency is improved considerably in the proposed CIGS PV device with BSF layer while maintaining the same absorber layer thickness in both cases. In Fig. 3, we can see that the efficiency of the cell with BSF layer is enhanced rapidly with increasing the absorber thickness from 0.1 to 0.8 μm and then is almost saturated. On the other hand, η of the PV device without BSF is changed somewhat beyond the CIGS absorber layer of 0.8 μm. According to the simulation results in Fig. 3, it can also be realized that the thickness should be required more than 3.0 μm to reach the maximum efficiency for the CIGS solar cell without BSF layer as compared to that of the cell with BSF layer. The selection of wider absorber will enhance the overall cost of the TFSC. Therefore, the optimal thickness of 0.8 μm is selected in both cases.

On the other hand, understanding how certain materials can create effective barriers despite seemingly unfavorable properties is crucial for the optimization of solar cell performance. It's crucial to take into account a few elements that can help the Sn_2_S_3_ BSF layer establish an appropriate barrier height in this case: Dangling bonds at the CIGS surface could be influenced by the Sn_2_S_3_ layer. The Sn_2_S_3_ layer can lower recombination rates by passivating these flaws, which helps to create an efficient barrier. However, quantum mechanical phenomena like tunneling might also be in operation. Even while Sn_2_S_3_ bulk characteristics could indicate a lower barrier, at the nanoscale, quantum phenomena can have a distinct impact on electron transport [56]. Additionally, engineering specific defects in the Sn_2_S_3_ layer can be a deliberate strategy to tailor its properties at the interface. Defects may act as trapping centers for charge carriers, contributing to the effective barrier.

#### 3.1.2 CIGS acceptor density impact on solar cell performance

**Fig. 4:**
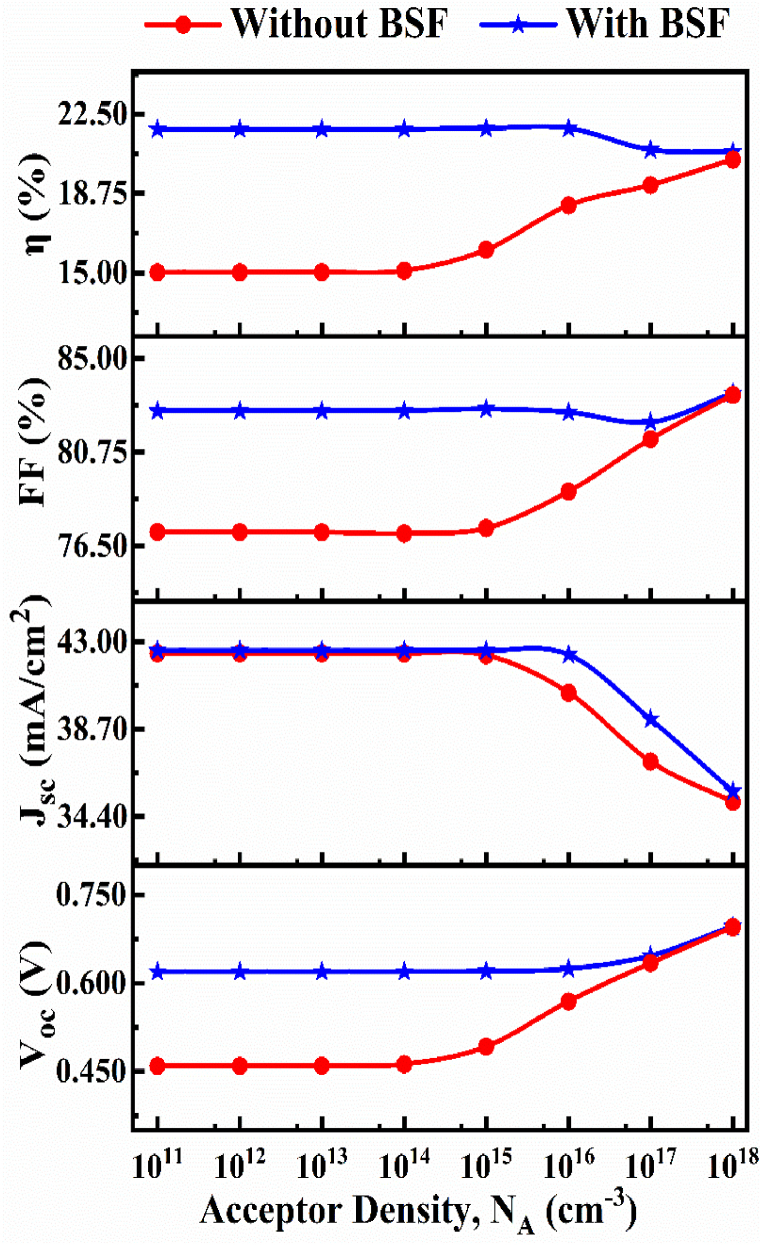
The impact of acceptor density of proposed CIGS solar cell structure with and without BSF.

The acceptor density (N_A_) of the CIGS layer has been changed from 1 × 10^11^ cm^−3^ to 1 × 10^18^ cm^−3^ due to find out optimized value of N_A_, at that time all other optimized value was constant in Table .1 and shown in Fig. 4. In this investigation period *V*_*OC*_ increases from 0.6197 to 0.6964 V, J_SC_ goes down from 42.56 to 35.63 mA/cm^2^, FF improved up from 82.62 to 83.42 %, and efficiency declined down from 21.79 to 20.7 %. After detail investigation we got the optimized acceptor density is 1 × 10^13^ cm^−3^ for CIGS absorber. In Fig. 4, it can be seen that the PV performance parameters are almost identical below the acceptor density of 10^15^ cm^−3^ for the CIGS absorber with and without BSF layer. On the other hand, the device outputs are changed at high doping density. However, it is very hard to achieve such high doping level in absorber materials experimentally. Therefore, in order to avoid complexity and to reduce expense, this simulation work is done with a reasonable and low absorber doping level of 1 × 10^13^ cm^−3^.

However, at higher acceptor density the decrease of photocurrent due to dominance of recombination of photo generated holes pairs. On the other hand the improved of Voc created from increased build-in-potential at lowering fermi-level at greater acceptor concentration and thereby larger band offset associate with inferior doping level at the buffer to absorber interface [57,58].

#### 3.1.3 The influence of bulk defects density of proposed CIGS solar cell

**Fig. 5:**
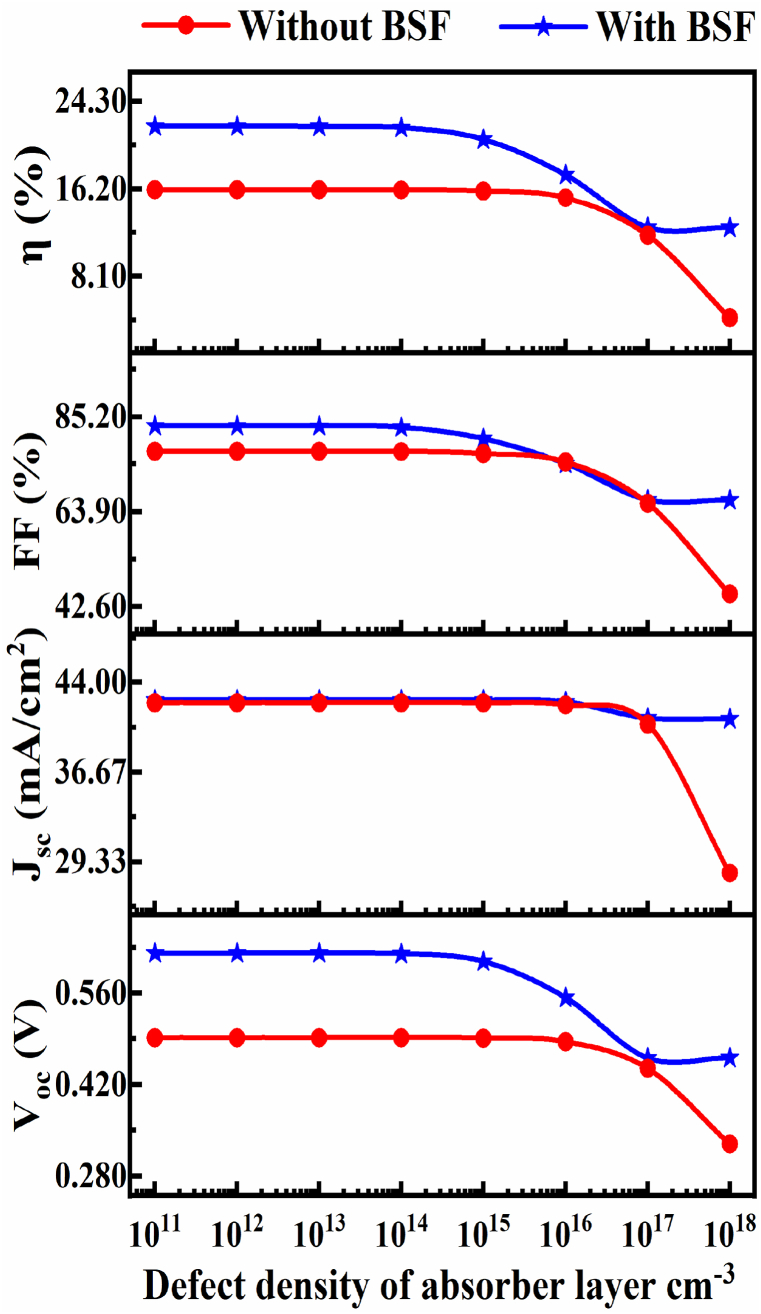
The impact of defect density with variation of CIGS absorber with and without BSF.

The impact of bulk defect density in the CIGS layer on the photovoltaic performances of the solar cell is investigated by changing the value 1 × 10^11^ cm^−3^ to 1 × 10^18^ cm^−3^, which is shown in Fig. 5. After investigation and optimization we got 1 × 10^14^ cm^−3^ is the optimized defect density for CIGS absorber. The value of V_OC_, J_SC_, FF, and PCE has been reduced while increasing the value of defect density. The enhancement of the defect increments the recombination rate, subsequently developing the reverse saturation current and due this result the overall PV performance are decayed [59].

#### 3.1.4 The influence of resistances on the proposed CIGS structure

**Fig. 6:**
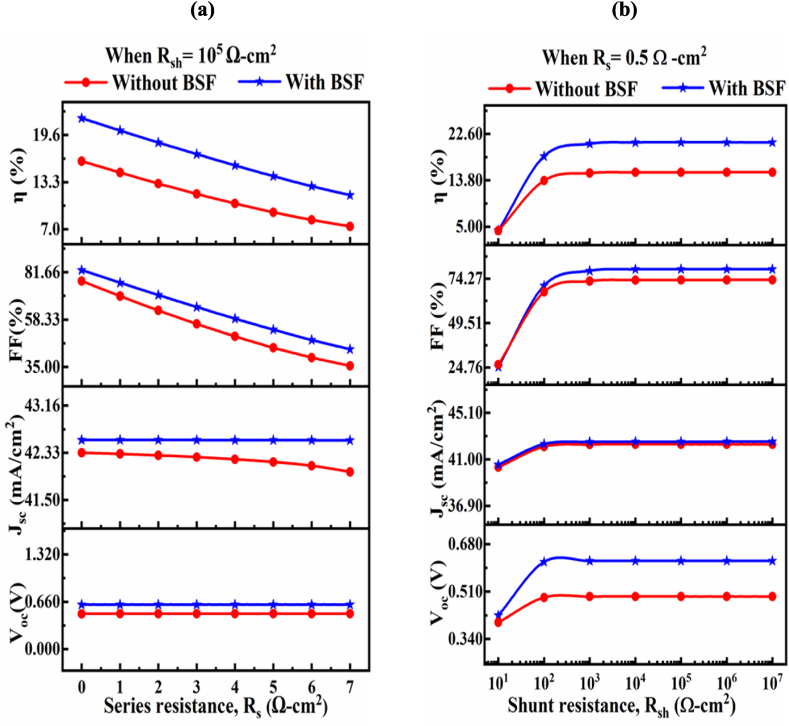
The impact of (a) series (R_s_), and (b) shunt (R_sh_) resistances on the characteristics of PV parameters of proposed structure with and without BSF.

The impact of series (R_s_) and shunt (R_sh_) resistances on the proposed solar cell structure with and without BSF is shown in Fig. 6. The all PV performance is influenced by parasitic resistances such as series (R_s_) and shunt (R_sh_) resistances. The series resistances of the structure create due to the bulk resistance and as well as the front and rear metallic resistance. On the other hand, the shunt (R_sh_) resistances created from different carrier recombination path due to manufacturing defect [60]. The cell structure of course has a lower R_s_ and greater R_sh_ due to improved highly efficient solar cell. Here, the shunt resistance, R_sh_, was set at 10^5^ Ω-cm^2^, while the value of the R_S_ was varied from 0 Ω-cm^2^ (ideal case) to 7 Ω-cm^2^. It is shown in Fig. 6(a), the Voc and Jsc are almost no changed with variation of Rs. However, it is noticed that the FF and PCE are extremely influenced by the increment of Rs. The FF is reduced from 81.66 to 35 % and PCE decreased from 21.83 to 11.54 % with the variation of Rs (0–7 Ω-cm^2^), respectively. Finally, it is shown that smaller Rs is create efficient cell structure, which is good agreement with the reputed report [60,61]. We have changed R_sh_ from 10 to 1 × 10^7^ Ω-cm^2^, while keeping the series resistance Rs constant at 0.5 Ω-cm^2^. The all PV parameter of the suggested solar cell is initially increased and then be inclined to saturated, which is shown in Fig. 6(b). For the suggested solar cell with the Sn_2_S_3_ layer, we showing that efficiency changed from 4.31 % to 21.00 % with respect to the rise in R_sh_, which is good agreement with the reputed literature [60].

#### 3.1.5 Impact of interface defect density on proposed solar cells

**Fig. 7:**
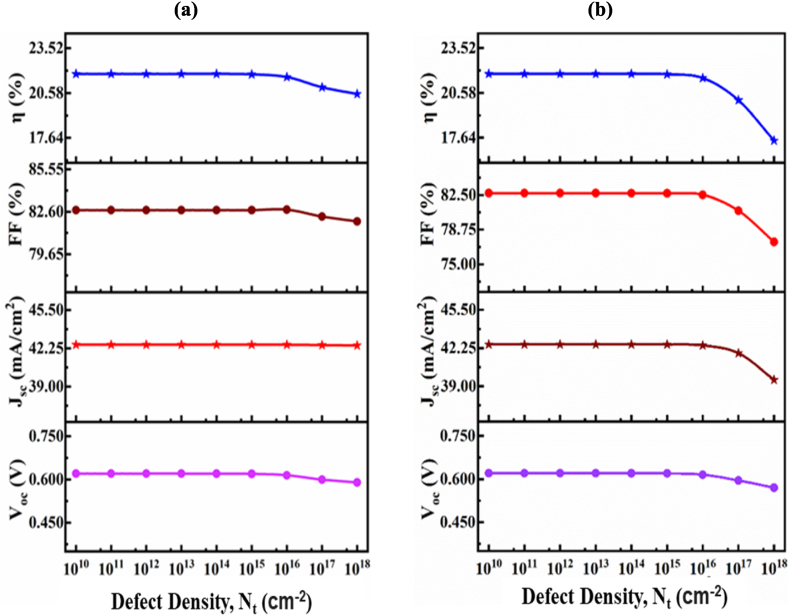
The influence of the interface defect density of (a) Sn₂S₃/CIGS and (b) CIGS/ZnS:In.

The impact of the interface defect density of Sn₂S₃/CIGS and CIGS/ZnS:In on the proposed solar cell structure is shown in Fig. 7(a) and (b). From figure, it is notice that how interface defect density (N_it_) impact the proposed cell structure performance. In the current study, the impact of the interface defect density at both ETL/CIGS and BSF/CIGS are investigated in details on the proposed structure. The opportunity of finding carriers at the interface increases with a rise in the interface state density (N_it_) between ETL/CIGS and BSF/CIGS. Consequently, carriers are more likely to be seized by their regional counterparts, as a result in low J_sc_ which reduction the PCE. On the other hand, the enhancement of the interface defect increments the recombination rate, subsequently developing the reverse saturation current and due to this result the overall PV performance are decayed on the proposed solar cell [61]. Finally, it is noticed that the optimized value of the defect density both of CIGS/ZnS:In and Sn₂S₃/CIGS interface is 10^10^ cm^−2^. In Fig. 7, the efficiency may appear similar between interface defect densities of 10^10^ cm^−2^ to 10^14^ cm^−2^, the considerations mentioned above, such as carrier transport, contact resistance, material stability, and manufacturing consistency, make the optimization of 10^10^ cm^−2^ a preferred choice in the CIGS solar cells. This optimization strategy aims to enhance the overall performance, reliability, and long-term viability of the solar cell technology.

The rectifying characteristics of a junction in highly efficient thin film solar cells can be evaluated by analyzing impact of interface quality. In order to realize the proposed CIGS heterojunction thin-film solar cells, the interface defect density is changed from 10^10^ to 1 × 10^18^ cm^−2^ to reveal the trend of the significance of the interface quality. The interface defects such as shallow or deep trap centers can be induced during fabrication of the thin-film solar cells [62]. The defects at interface could act as charge trap centers for photo-excited electrons, which affect the electrons injection and electron-hole recombination at the interface [63]. In Fig. 7, it can be observed that the power conversion efficiency changes insignificantly for the interface defect density below 10^16^ cm^−2^. This unaffected region may be attributed to the shallow defect states located at the interface. The shallow defect dominates to slow down the charge transport and electron-hole recombination at the interface. In addition, the series resistance may also be increased significantly with the increment of interface defect [64]. The outputs of the solar cell should be degraded by increasing series resistance. However, the series resistance doesn't have great effect on short-circuit current density J_sc_, and thus resulted in the almost constant efficiency with variation of defect density.

#### 3.1.6 Operating temperature impact

**Fig. 8:**
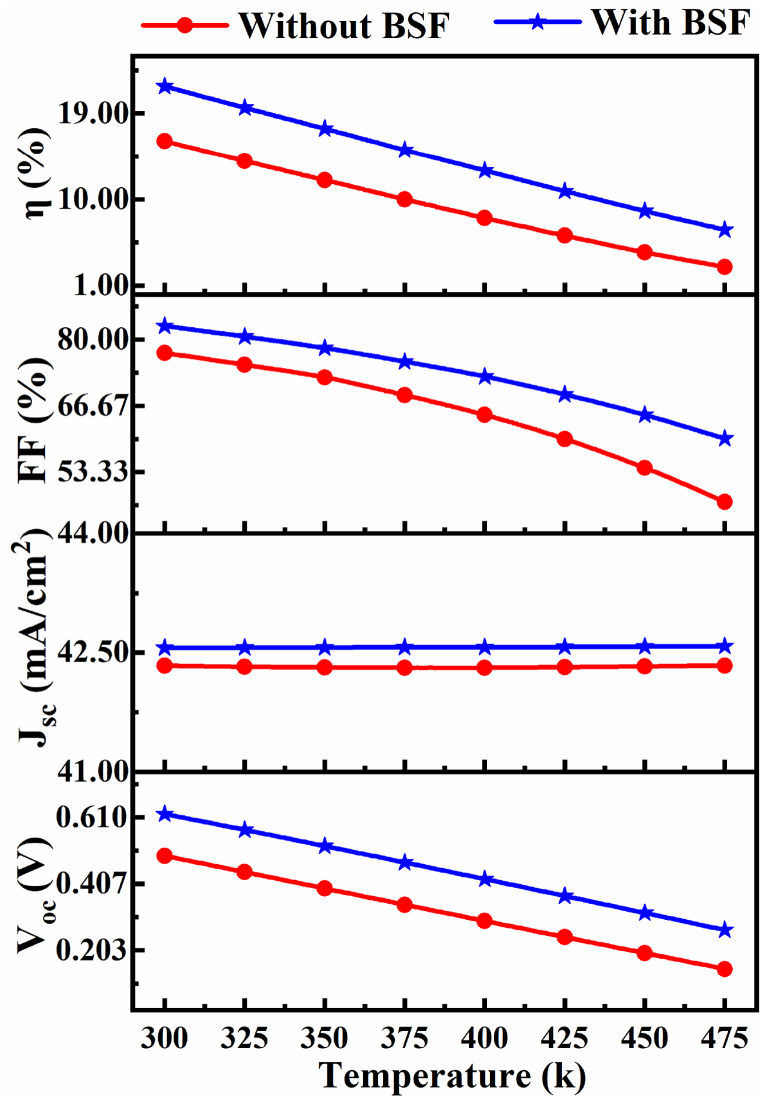
Temperature (T) effect of proposed CIGS solar cell with and without BSF.

The temperature (T) effect is investigated to compare the performance of the proposed CIGS solar cell with and without BSF and shown in Fig. 8. Furthermore the T was changed from 300 K to 475 K to investigate the correlation between T and PCE of the proposed CIGS cell. From Fig. 8 it is clearly shown that V_OC_, FF and PCE are drastically decline with increasing temperature with and without BSF, while the J_SC_ values remain almost unchanged. Higher temperatures lead to poorer efficiency because of temperature-dependent factors for example electron-hole mobility, carrier concentrations, and energy band gaps, which is good agreement with the reputed works [59].

## 4 Output performance of proposed CIGS solar cells

### 4.1 J-V characteristics of proposed CIGS solar cells

**Fig. 9:**
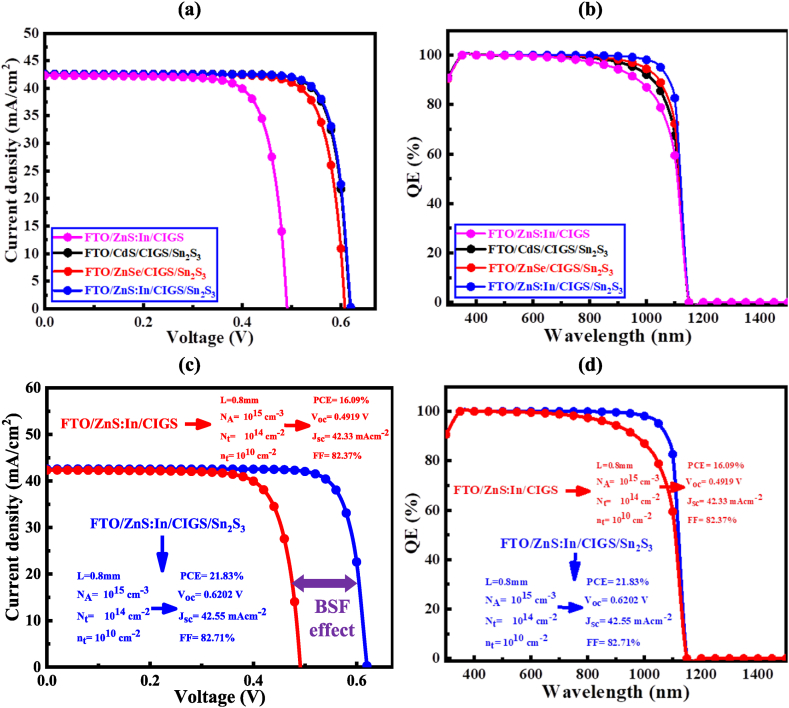
(a) J-V characteristics with various ETL layers, (b) Quantum efficiency with various ETL layers, (c) J-V characteristics with and without optimized ETL layers, and (d) Quantum efficiency with and without optimized ETL layers of proposed CIGS solar cells.

The current-voltage (J-V) and quantum efficiency (QE) characteristic curves of the proposed CIGS solar cells are shown in Fig. 9. It is noticed that Fig. 9(a) and (b) shows the J-V and QE due to various ETL. On the other hand, Fig. 9(c) and (d) shows the J-V and QE due to optimized ETL with and without BSF. In the proposed CIGS solar cell structure, the employing of 0.8 μm thick absorber layers provides the better performance than the traditional CIGS solar cells. The optimized photovoltaic properties with 0.8 μm thick absorber layers are shown in Table 3 with and without BSF. It is confirmed that after simulating the PV parameters, ZnS:In buffer layer shows superior performance compared to the other buffer. Our optimized structure shows the voltage (V_OC_) 0.4919 V (0.62202 V), short-circuit current density (J_SC_) 42.33 mA/cm^2^ (42.55 mA/cm^2^), fill factor (FF) 82.30 % (82.71 %), and power conversion efficiency (PCE) 16.09 % (21.83 %) without (with) BSF, respectively. It is interesting to notice that this result also supports the lattice mismatch value, which is shown in Table 5 and discuss in section 4.1.3 in this manuscript.

**Table 3:** Optimized result of proposed CIGS cell with and without BSF.

Cell type	CIGS depth (μm)	Sn₂S₃ depth (μm)	J_SC_ (mA/cm2)	Voc(V)	FF (%)	η (%)
Conventional	0.8	–	42.33	0.4919	77.3	16.09
This work	0.8	0.05	42.55	0.6202	82.71	21.83

**Table 4:** Influence of back-surface field (BSF) in similar research.

S/N	Field of research	Absorber	BSF	η without BSF(%)	η with BSF (%)
1	Experimental	Si	ZnS	6.40	11.02 [65]
2	”	Si	Al	12.96	13.75 [66]
3	”	CIGS	MoSe_2_	9	14 [65]
4	Theoretical	CZTS	CZTS	12.05	14.11 [67]
5	”	CZTSSe	SnS	12.30	17.25 [68]
6	”	CIGS	Si	16.39	21.30 [12]
**7**	**”**	CIGS*	**Sn**_**2**_**S**_3_*	**15.94**	**20.77 (ETL: ZnSe)**
**8**	**”**	CIGS*	**Sn**_**2**_**S**_3_*	**15.22**	**21.70 (ETL: CdS)**
**9**	**”**	CIGS**	**Sn**_**2**_**S**_3_**	**16.09**	**21.83 (ETL:ZnS:In)**

Note: [*] indicates proposed cell and [**] indicates optimized cell.

**Table 5:** Lattice mismatch at different ETLs.

Layer	Lattice Parameters			Lattice Mismatch
	a (Å)	b (Å)	c (Å)	
**CIGS**	5.79	5.79	11.47	
**CdS**	4.11	4.11	6.72	33.79 %
**ZnSe**	4.00	4.00	6.57	36.56 %
**ZnS:In**	5.39	5.39	5.39	7.15 %

Quantum efficiency (QE) is a measurement of how successfully a solar cell or other light-sensitive device turns absorbed light into electrical current. There are two main ways to analyze quantum efficiency: internal quantum efficiency (IQE) and external quantum efficiency (EQE). The quantum efficiency (QE) value of a solar cell indicates how much current the PV cell can produce when exposed to photons of a particular wavelength. The external quantum efficiency (EQE) is a measurement of the number of electrons that can be extracted from a PV device per incident number of photons [57]. The device's QE rises as absorber thickness increases because a larger layer collects more photons. Each material may absorb photons within a specific wavelength range [55].

At a certain wavelength, all the QE curves begin to decline towards zero, Fig. 9(b) and (d) shows the QE curves at a wavelength range of 300–1500 nm of the base CIGS and modified structures at different ETLs, with and without the BSF layer. All curved lines begin to reduce until they have zero percent QE at specific wavelengths for the reason that at a particular wavelength range of the visible light spectrum the materials absorb photons. Moreover, it is observed that the EQE at the wavelength below 500 nm is high. This may be due to capture of more photons by the absorber layer in the short wavelength range. Thus, this absorbed incident photons would create a sufficient number of electron-hole pairs. The behavior of the EQE at the short wavelength range is similar to the simulation results reported in the previous works [[69], [70], [71]]. However, Table 4 display the impact of BSF of the related similar research.

#### 4.1.1 The capacitance-voltage (C–V) analysis

**Fig. 10:**
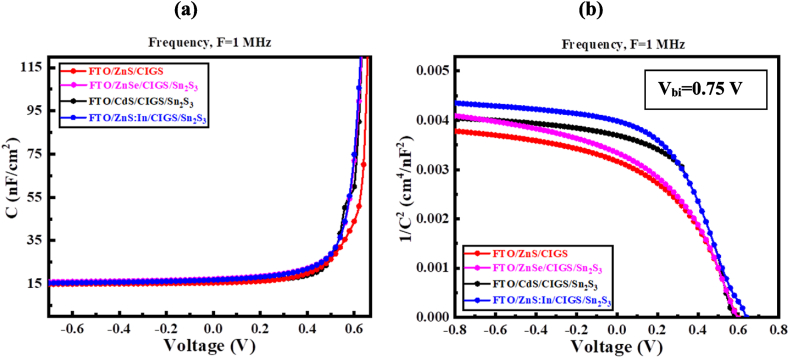
Impact of (a) C–V and (b) 1/C^2^–V curve on proposed CIGS solar cell.

To check the consistency of the analysis, the capacitance-voltage (C–V) analysis was carried out at a frequency of 1 MHz. The depletion and diffusion capacitances are connected to the p-n junctions. Diffusion capacitance predominates at forward bias voltage, whereas depletion capacitance is bigger in magnitude at reverse bias voltage. As shown in Fig. 10(a), the capacitance of a p-n junction solar cell is 15 nF cm^−2^ at the zero bias voltage. When the polarization potential is increased at a specific frequency and voltage level the capacitance increases exponentially. This tendency is demonstrated by how insensitive the absorber traps are to all frequencies. Effective traps do not contribute to reducing the effective charge at the reverse bias, which shows lowers capacitance [53,72].

The Mott-Schottky plot of the PV cell has been illustrated in Fig. (b). The intersection of the 1/C2 plot with the voltage axis yields the flat-band potential of the proposed SC [53]. The p-type CIGS layer and the space charge area are both primarily occupied by holes, according to the plot's negative slope. As a result of exposure to sunlight, PGCs may be the source of the high carrier density in the CIGS layer. The localized deep states in the absorber layer may be causing the marginal deviation for 1/C^2^. The main effect is brought on by the modulation of the majority carriers, not by the influence of the deep states [53,72]. Since the voltage of reverse bias and inverted square capacitance have a linear relationship, the heterojunction is abrupt in nature, and the built-in potential (V_bi_) values are determined when the straight line intersects the voltage axis at (1/C^2^ = 0). In our proposed structure the average V_bi_ = 0.75 V which is well matched various reputed works [73,74].

#### **4.1.2** Lattice mismatch

The efficiency improvement may also be supported by determining the lattice mismatch between the absorber and buffer layer using equation (1) [53]:

(1) $$\delta =2\left|a_{s}-a_{e}\right|/\left(a_{s}+a_{e}\right)$$

Where, δ, $a_{s}$, and $a_{e}$ are lattice mismatch, substrate and epitaxial thin film lattice constant respectively.

The lattice constant for the various materials has been calculated based on earlier research [75,76]. Lattice mismatch between CIGS and ZnS is 7.15 %, which is less than that of the ETLs used in earlier investigations. The presence of defect and interface defect density, which causes carrier losses and a decrease in output voltage, is the main cause of non-radiative recombination [77]. The interface defect density and recombination loss are greatly reduced by the smaller lattice mismatch between ZnS and CIGS. As a result, the performance characteristics are greatly improved by the addition of the ZnS ETL layer to the CIGS structure. Because of the increase in minority carrier recombination, the Al/FTO/ZnS:In/CIGS/Ni reference structure exhibits reduced V_OC_ despite the absence of BSF. As a result, the performance metrics of the device have been impacted.

## 5 Conclusions

The present study reports on designing CIGS based solar cell with various novel buffer layer. Furthermore, a detailed investigation was deliberate into the effects of absorber and buffer thickness, defect density, doping concentration, resistances, and temperature on the proposed cell as a optoelectronic output characteristics using SCAPS-1D. In the beginning, the toxic cadmium Sulfide (CdS) buffer of the proposed structure FTO/CdS/CIGS/M (M = Ag, Au, Co, Cr, Cu, Mo, Sn, and Ni) has been replaced by various non-toxic buffers: such as zinc selenide (ZnSe), and indium (In)-doped Zinc Sulfide (ZnS:In). After investigated we recommend the use of Ni as the best back metal and ZnS:In is the superior buffer in the proposed solar cells. Then we added a 0.05 μm thin novel Sn₂S₃ BSF layer with optimized structure (Al/FTO/CdS/Sn₂S₃/CIGS/Ni), which significantly reducing the thickness as well as cost of the absorber materials but enhance the PCE from 16.09 to 21.83 %. This work provides a design guideline on how to utilize an ultra-thin Sn₂S₃ layer as a BSF in conventional CIGS solar cells to enhance overall performance with a significant reduction in absorber material cost. Present findings suggest that the possibility of fabricating a low-cost CIGS-based thin-film solar cell (TFSC) near future towards a high efficiency of ≈22 %.

## Data availability

The data that support the findings of this study are available from the corresponding author upon reasonable request.

## CRediT authorship contribution statement

**Md. Ferdous Rahman:** Writing - review & editing, Writing - original draft, Visualization, Validation, Supervision, Software, Resources, Project administration, Methodology, Investigation, Formal analysis, Data curation, Conceptualization. **Md. Kamrul Hasan:** Writing - original draft, Software, Methodology, Formal analysis, Data curation, Conceptualization. **Mithun Chowdhury:** Writing - original draft, Software, Resources, Formal analysis, Data curation. **Md. Rasidul Islam:** Writing - original draft, Validation, Software, Formal analysis. **Md. Hafijur Rahman:** Writing - original draft, Visualization, Validation, Resources, Methodology. **Md. Atikur Rahman:** Writing - original draft, Validation, Methodology, Formal analysis. **Sheikh Rashel Al Ahmed:** Writing - original draft, Software, Resources, Formal analysis. **Abu Bakar Md. Ismail:** Writing - original draft, Software, Resources, Formal analysis. **Mongi Amami:** Writing - original draft, Validation, Software, Resources, Funding acquisition. **M. Khalid Hossain:** Writing - original draft, Visualization, Methodology, Formal analysis. **Gamil A.A.M. Al-Hazmi:** Writing - original draft, Validation, Methodology, Funding acquisition.

## Declaration of competing interest

The authors declare that they have no known competing financial interests or personal relationships that could have appeared to influence the work reported in this paper.
