# Supplementary material for: A qualitative Design and optimization of CIGS-based Solar Cells with Sn2S3 Back Surface Field: A plan for achieving 21.83 % efficiency
Source: Heliyon. 2023 Nov 25;9(12):e22866. doi: 10.1016/j.heliyon.2023.e22866 (PMC10730753; doi:10.1016/j.heliyon.2023.e22866)
Supplement: Multimedia component 1 [file mmc1.docx]

**Form to confirm authorship changes for Heliyon**

This form must be signed by all authors when there is a change in authorship which includes changes to any of the following items: author name(s), order of the authors, the corresponding author(s), the addition of authors, the removal of authors and changes in affiliation. By personally signing this note, all authors confirm that: I) the changes are in accordance with their scientific contribution, II) they agree with all the changes and III) confirm that the authorship list conforms to the authorship criteria outlined on Heliyon’s ethics page. IV) it is the responsibility of the corresponding author to get the signature from all co-authors accepting the change. In case of any ethic violation/malpractice in the signature, the corresponding author is accountable. The completed form should be returned along with the final/revised manuscript to proceed further with the manuscript. Manuscripts for which incomplete forms have been submitted will be rejected within 5 working days. Any disputes on the authorship list and contributions need to be resolved by the involved scientists and Heliyon will only proceed with the evaluation of the manuscript once we receive confirmation, through this form, that such an agreement between the authors has been reached.

Manuscript number: HELIYON-D-23-34419R2

Article title: A qualitative Design and optimization of CIGS-based Solar Cells with Sn_2_S_3_ Back Surface Field: a plan for achieving 21.83% efficiency

Complete new author list: Md. Ferdous Rahman, Md. Kamrul Hasan, Mithun Chowdhury, Md. Rasidul Islam, Md. Hafijur Rahman, Md. Atikur Rahman, Sheikh Rashel Al Ahmed, Abu Bakar Md. Ismail, Mongi Amami, M. Khalid Hossain, Gamil A. A. M. Al-Hazmi

Date: 11.21.2023

| **SL** | **First name** | **Last name** | **Order change (Y/N)** | **Addition /Deletion** | **Change in Author name** | **Affiliation Change (Y/N)** | **Reason for the change** | **Signature** |
| --- | --- | --- | --- | --- | --- | --- | --- | --- |
| **01** | Md. Ferdous | Rahman | **N** |  |  | **N** |  | 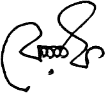 |
| **02** | Md. Kamrul | Hasan | **N** |  |  | **N** |  | 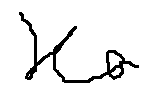 |
| **03** | Mithun Chowdhury |  | **N** |  |  | **N** |  | 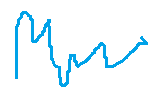 |
| **04** | Md. Rasidul | Islam | **N** |  |  | **N** |  | 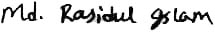 |
| **05** | Md. Hafijur | Rahman | **N** |  |  | **N** |  | 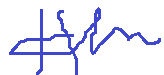 |
| **06** | Md. Atikur | Rahman | **N** |  |  | **N** |  | 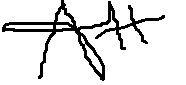 |
| **07** | Sheikh Rashel | Al Ahmed | **N** |  |  | **N** |  | 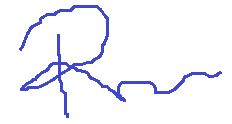 |
| **08** | Abu Bakar Md. | Ismail | **N** |  |  | **N** |  |  |
| **09** | Mongi | Amami | N |  |  | N |  | 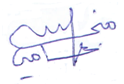 |
| **10** | M. Khalid | Hossain | N |  |  | N |  | 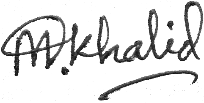 |
| **11** | Gamil A. A. M. | Al-Hazmi |  | Addition |  |  | Revise manuscript # HELIYON-D-23-34419R2 prepare the reply letter, contributed reagents, materials, analysis tools or data; and wrote the paper. | 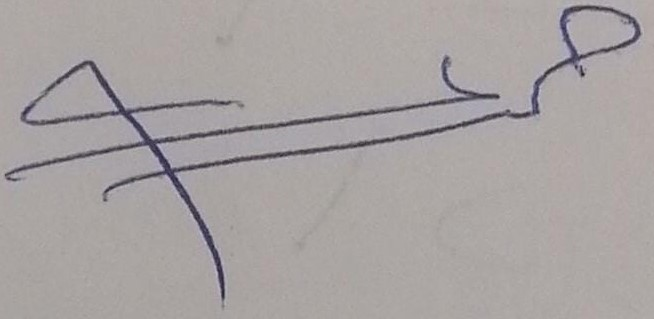 |

Sincerely,

**Dr. Md. Ferdous Rahman** (Corresponding author)

Associate Professor and Head of the Department

Department of Electrical and Electronic Engineering

Begum Rokeya University, Rangpur 5400, Bangladesh

E-mail: ferdousapee@gmail.com

ORCID: 0000-0002- 0090-2384
